# Efficacy and safety of tumor-treating fields combined with concurrent chemoradiotherapy for newly diagnosed glioblastoma patients

**DOI:** 10.1097/MD.0000000000048383

**Published:** 2026-04-24

**Authors:** Zixuan Wang, Zhenyuan Jiang, Dan Zong, Huanfeng Zhu, Guannan Zou, Jingshu Xu, Wenjie Guo, Yizhi Ge, Xiao Wang, Jiamin Yu, Xia He

**Affiliations:** a Department of Radiation Oncology, Affiliated Cancer Hospital of Nanjing Medical University, Jiangsu Cancer Hospital and Jiangsu Institute of Cancer Research, Nanjing, China; b Postgraduate College, NanJing Medical University, Nanjing, China; c Jiangsu Key Laboratory of Innovative Cancer Diagnosis & Therapeutics, Nanjing, China; d Postgraduate College, Xuzhou Medical University, Xuzhou, China; e Department of Oncology, The Second Affiliated Hospital of Nantong University, Nantong First People’s Hospital, Medical School of Nantong University, Nantong, China; f Department of Radiology, The Affiliated Cancer Hospital of Nanjing Medical University & Jiangsu Cancer Hospital & Jiangsu Institute of Cancer Research, Nanjing, China.

**Keywords:** efficacy, glioblastoma, safety, tumour treating fields

## Abstract

**Background:**

Tumour treating fields (TTFields) have demonstrated efficacy in extending survival in the temozolomide maintenance therapy phase. Consequently, the purpose of this study is to investigate the safety and efficacy of TTFields in combination with concurrent chemoradiotherapy for newly diagnosed glioblastoma (GBM) patients, as well as analyzing the prognostic factors for these patients, can provide valuable data to guide the clinical application of TTFields.

**Methods:**

This study retrospectively analyzed the clinical treatment of 68 GBM patients receiving the TTFields, admitted to the Department of Radiation Oncology at Jiangsu Cancer Hospital from September 2021 to October 2024. Progression-free survival (PFS) curves and overall survival were constructed using the Kaplan–Meier method. A Cox proportional hazards regression model was used to assess the effect of TTFields and to account for confounding factors.

**Results:**

Among the 68 GBM patients included in the study, the median PFS was 16.4 months (95% CI: 13.3–19.5), the median overall survival was 28.8 months (95% CI: 20.6–37.0) for all 68 patients. The objective response rate was 64.7% (44/68), and the disease control rate was 94.1% (64/68). Multivariate analysis revealed that surgery type and compliance rate with TTFs were favorable prognostic factors. Mild to moderate skin toxicity underneath the transducer arrays occurred in 51.4% of patients.

**Conclusion:**

In our final analysis of single-center Chinese patients with newly diagnosed GBM, concurrent chemoradiotherapy with TTFields resulted in promising PFS outcomes that warrant validation in propensity-matched or randomized studies.

## 1. Introduction

The incidence of glioblastoma (GBM) in China is steadily increasing, with recent data indicating an incidence rate of approximately 8.8 per 100,000 in 2022.^[1]^ Among brain tumors, GBM, which is the most malignant form, accounts for approximately 45.6% of cases, with an almost 100% recurrence rate. The standard treatment for GBM includes surgical resection, radiotherapy, and chemotherapy. Despite undergoing the standard treatment, patients typically survive for only 12 to 18 months after diagnosis.^[2]^ The extent of tumor resection is widely regarded as a crucial factor in determining patient prognosis. In 2005, Stupp et al added temozolomide to radiotherapy and initiated temozolomide maintenance therapy, resulting in an increase in overall survival (OS) from 12.0 to 14.6 months, marking the beginning of temozolomide treatment for GBM.^[3]^ Radiotherapy is a very critical part. Timely radiotherapy after surgery is of great significance for effectively prolonging the survival time of patients. Unfortunately, in nearly a decade of research in the field of GBM, the improvement in OS has been minimal. The use of a tumor-treating fields (TTFields) for delivering low-intensity (2 V/cm) and intermediate-frequency (100–300 kHz) alternating electric fields is thought to reduce tumor cell proliferation and promote cell death by altering the cell cycle of cells in mitosis.^[4]^ The EF-14 trial, a milestone significance clinical trial reported in 2017, showed that the addition of TTFields after radiotherapy improved progression-free survival (PFS) by 2.7 months (4.0 vs 6.7 months) and OS by 4.9 months (16.0 vs 20.9 months) in newly diagnosed GBM patients.^[5]^ The Chinese Food and Drug Administration also formally approved TTFields for newly diagnosed GBM in 2020. Additionally, 2 significant studies have confirmed the synergistic effects of TTFields in combination with chemoradiotherapy.^[6,7]^ Therefore, trimodal therapy involving temozolomide-based concurrent chemoradiotherapy plus TTFields after maximum safe tumor resection is safe, and its efficacy is maximized. This article is the first to report the use of TTFields combined with concurrent chemoradiotherapy for the treatment of GBM patients in China. This study aimed to evaluate the efficacy and safety of TTFields combined with concurrent chemoradiotherapy as a first-line treatment in Chinese newly diagnosed GBM patients, and provide favorable guidance and basis for future clinical medication.

## 2. Materials and methods

### 2.1. Patients

**Figure 1.**
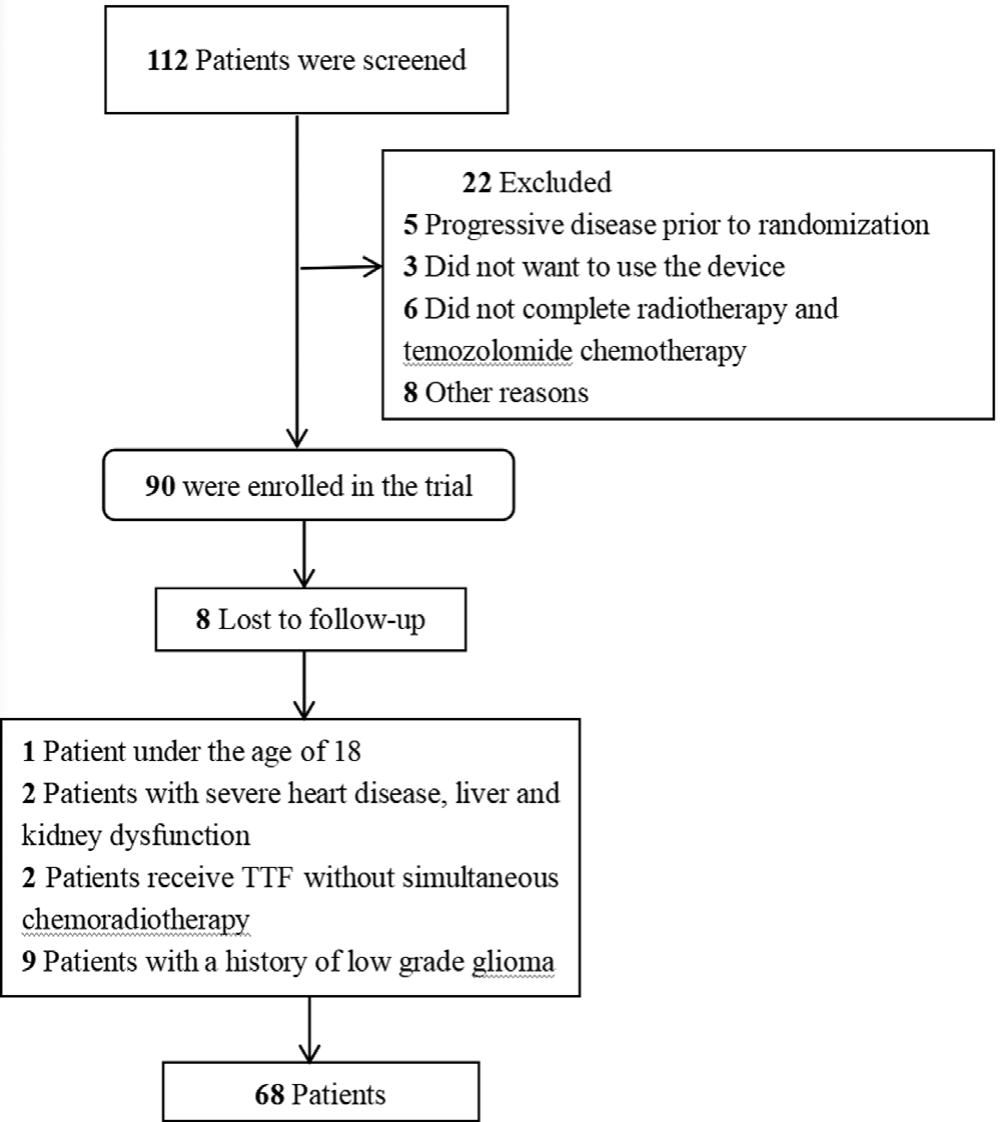
Flowchart of patient enrollment in this study. (A) Progression-free survival; (B) overall survival.

All patients eligible for this study met the following criteria: aged between 18 and 78; had undergone maximal safe surgery and had histologically confirmed supratentorial GBM (World Health Organization grade IV); adequate bone marrow, liver, and renal function; and were postoperatively treated with TTFields concurrent with standard concomitant chemoradiotherapy with temozolomide, with TTFields use for ≥1 month. After strict exclusion and screening, a total of 68 patients met the admission criteria of this study. The specific admission flowchart is shown in Figure 1. This study was approved by the Ethics Committee of Jiangsu Cancer Hospital (approval number: 2022-028).

### 2.2. TTFields instruments and usage

The TTFields device used in this study received marketing approval from the Food Drug Administration in 2020. The TTFields device used in this study was the NovoTTF-200A system (Novocure GmbH, Root, Switzerland), approved by the National Medical Products Administration of China in May 2020 for the treatment of newly diagnosed and recurrent glioblastoma. The NovoTTF 200A system comprises 2 main components: an electric field generator (preset to 200 kHz) and insulated transducer arrays. The device treatment kit includes a plug-in power supply, portable battery, battery rack, battery charger, connecting cables, and carrying case. The transducer array layout for individual GBM patients can be optimized based on measurements obtained from magnetic resonance imaging data, depending on the head size and tumor location. TTFields devices were worn for at least 5 days prior to the start of radiation therapy for at least 42 days. Patients were instructed to replace the transducer array every 2 to 3 days and to maintain scalp hygiene through shaving and cleaning. If the array is displaced, it should be moved back to its original position to ensure optimal targeting of the tumor bed. Device support specialists reinforce compliance management by regularly downloading usage data through log analysis. Patients are actively encouraged to prolong their compliance with TTFields treatment, while doctors actively deal with adverse skin reactions resulting from treatment. The electrode array should be maintained, the device should be powered off before daily radiotherapy and placed outside the treatment area, to decrease skin irritation extending the duration of TTFields.

### 2.3. Radiotherapy programme design

The clinical target volume was expanded by 2 cm from the gross tumor volume, and the necessary aa was adjusted. The planning target volume (PTV) was expanded 3 mm from the clinical target volume, with a prescribed dose of 2 Gy per fraction for a total of 30 fractions. The normalization criterion ensured that 95% of the PTV received 100% of the prescribed dose. The target area outlining and plan design were performed by Eclipse 15.6. The radiotherapy techniques employed included fixed-field intensity-modulated radiotherapy and volumetric arc intensity-modulated radiotherapy. Given the challenge of scalp adverse effects resulting from the combination of TTFields and radiotherapy, careful monitoring of the scalp dose to minimize toxicity is paramount. As such, the scalp serves as the limit for dose delivery to the organ at risk during target areas. The scalp structure was defined as a thickness of 5 mm from the skin surface extending beyond the level of the foramen magnum of the occipital bone. The criteria for scalp dose limits were as follows: $D_{20\;cm^{3}}$ < 50 Gy and $D_{30\;cm^{3}}$ < 40 Gy. If necessary, priority was given to PTV coverage beyond the scalp dose limits.

### 2.4. Chemotherapy protocol

All patients underwent postoperative radiotherapy combined with temozolomide (75 mg/m^2^) synchronous chemotherapy followed by 6 to 12 cycles of temozolomide (150–200 mg/m^2^) adjuvant chemotherapy.

### 2.5. Evaluation of effectiveness and safety

The extent of tumor resection was assessed based on the patients’ cranial magnetic resonance imaging (including scanning and enhancement) performed within 24 to 72 hours postoperatively, which served as the baseline postoperative imaging data for gliomas. The evaluation of GBM treatment response followed the Response Assessment in Neuro-Oncology criteria.^[8]^ Moreover, according to the Macdonald criteria, a ≥25% increase in lesions within the contrast-enhanced region indicated tumor progression. Efficacy outcomes (complete remission, partial remission, stable disease, progressive disease) were determined by a specialist radiologist based on pre- and posttreatment imaging. Magnetic resonance imaging enhancement was conducted every 3 months following the initiation of TTFields or upon clinical assessment of tumor progression. The primary endpoint was the median PFS and OS time, and the secondary endpoints were the objective response rate, disease response rate. The patients were assessed for disease efficacy via Kaplan–Meier analysis to calculate PFS and OS time. Adverse events were recorded according to the Common Terminology for Common Adverse Events version 5.0 criteria (CTCAE 5.0) and the TTField-related dermatological adverse event (dAE) grading criteria. Safety monitoring involved weekly routine blood and biochemical monitoring, with a focus on adverse events during treatment. In cases of abnormalities, adverse event assessments were conducted, and symptomatic treatment was administered. dAEs were assessed at least once a month by trained personnel, with each event evaluated in relation to TTFields based on localized skin irritation, and symptomatic treatment was given by dermatologists.

### 2.6. Compliance assessment

Treatment compliance data reports were collected monthly, utilizing reports generated from the use of the NovoTTF-200A therapeutic device. Compliance was calculated as the percentage of TTFields patches used within 24 hours per day after placement.

### 2.7. Follow-up

Routine follow-up included weekly blood tests and blood biochemistry tests, cranial magnetic resonance scanning and enhancement examination every 3 months, and dAEs were assessed monthly by a professional physician. Regular telephone follow-ups were also conducted during this period to closely monitor the patient’s condition.

### 2.8. Statistical methods

Data were analyzed using SPSS Statistics 28.0 software (IBM Corporation, Armonk). Survival analysis was performed using the Kaplan–Meier method, and the log-rank test was used for comparison. *P* < .05 indicated a statistically significant difference. The Cox proportional hazards model was used to analyze various factors, including sex, tumor resection status, use of oral anlotinib, O6-methylguanine-DNA-methylguanine (MGMT) promoter methylation status, telomerase reverse transcriptase (TERT) promoter mutation status, 1p19q status, and TTF treatment adherence. A *P* value < .05 indicated a statistically significant difference. Plans are under way to perform a multi-center propensity-score-matched comparison using a contemporaneous cohort not treated with TTFields; these results will be reported separately.

## 3. Results

### 3.1. Study population

A total of 68 patients with newly diagnosed GBM admitted to the Radiotherapy Department of the Affiliated Cancer Hospital of Nanjing Medical University between September 2021 and December 2023 were enrolled for our study. The median age of all patients was 55 years (range 28–78 years). Among them, 54.4% were male, and 45.6% were female. About 36.8% had undergone gross total reset, while 73.5% had undergone a standard Stupp regimen of simultaneous oral anlotinib (8 mg/d). A total of 83% of these patients demonstrated a compliance rate > 80%. All patients underwent surgery with the standard Stupp regimen. Additional basic clinical information is shown in Table 1.

**Table 1:** Patient baseline characteristics and treatments.

Characteristics	No. (%) of patients
Sex	
Men	37 (54.4%)
Women	31 (45.6%)
Extent of resection	
Gross total resection	25 (36.8%)
Partial resection	43 (63.2%)
Biopsy	0 (0%)
Anlotinib plus TTFields and radiochemotherapy	
Yes	50 (73.5%)
No	18 (26.5%)
MGMT promotor region methylation status	
Methylated	20 (29.4%)
Unmthylated	23 (33.8%)
Unknown	25 (36.8%)
IDH status	
Wild-type	37 (54.4%)
Mutated	11 (16.2%)
Unknown	20 (29.4)
TERT promoter mutation status	
Methylated	19 (27.9%)
Unmthylated	11 (16.2%)
Unknown	38 (55.9%)
Tumor tissue chromosomes 1p and 19q	
Codeletion	4 (5.9%)
Retained	21 (30.9%)
Unknown	43 (63.2%)
Karnofsky performance score	
≥60	65 (95.6%)
<60	3 (4.4%)
Tumor-treating fields therapy	
Duration, median (range)	
≥18 h/d	83% (50–95%)
Completed standard Stupp protocol	
Yes	68 (100%)
No	0 (0%)

GBM = glioblastoma, IDH = isocitrate dehydrogenase, MGMT = O6-methylguanine-DNA-methyltransferase gene, TERT = telomerase reverse transcriptase, TTFields = tumor-treating-fields.

### 3.2. Follow-up and patient survival

**Figure 2.**
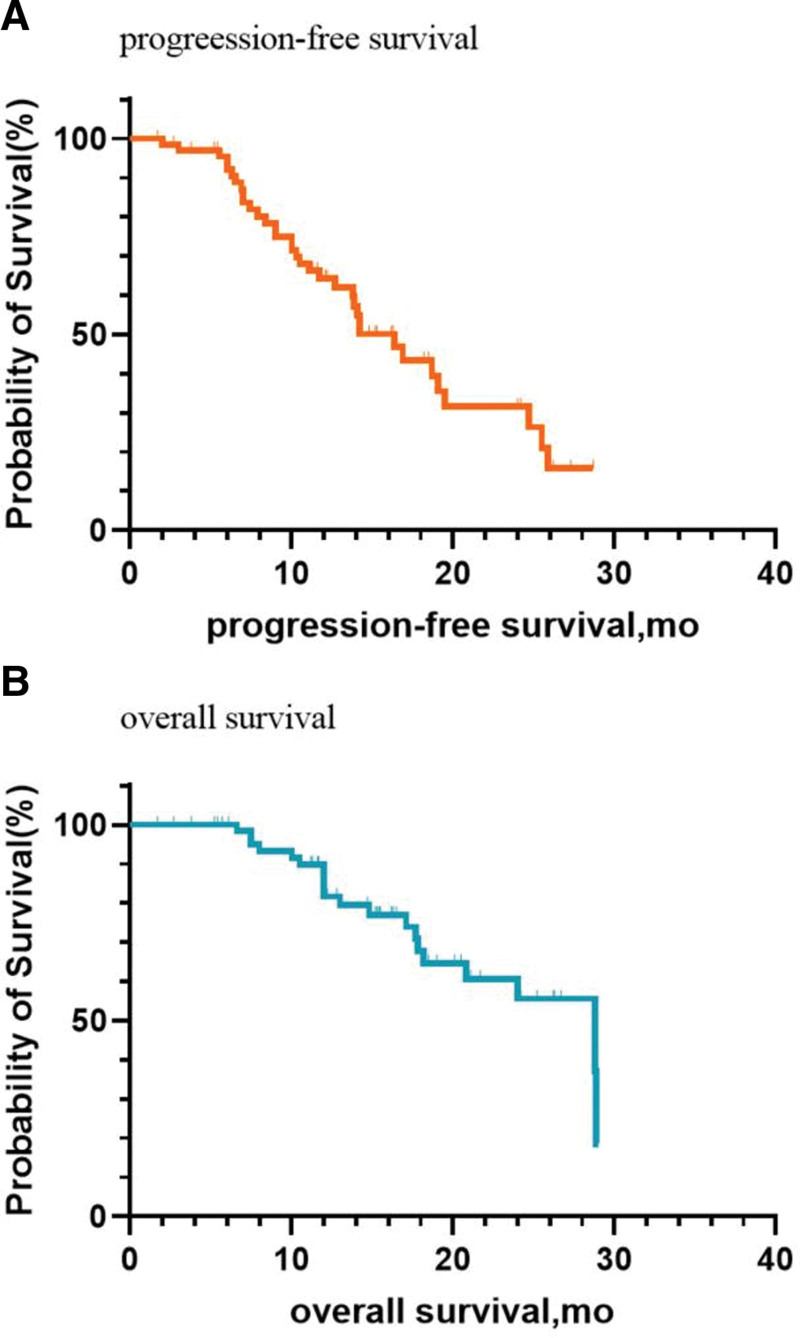
Kaplan–Meier survival curve for GBM patients. GBM, glioblastoma.

Follow-up concluded in March 2024, with a median follow-up duration of 14.7 months. As of the last follow-up date, 33 of the 68 patients were stable, and 35 had progressed (20 died). The median PFS was 16.4 months (95% CI: 13.3–19.5) for all 68 patients, the Kaplan–Meier survival curve of the patients is shown in Figure 2A. The median OS was 28.8 months (95% CI: 20.6–37.0) for all 68 patients, the Kaplan–Meier survival curve of the patients is shown in Figure 2B. The disease response rate was 94.1% (64/68), and the objective response rate was 64.7% (44/68) after using TTFields combined with concurrent chemoradiotherapy.

### 3.3. Prognostic factors and survival rate

**Figure 3.**
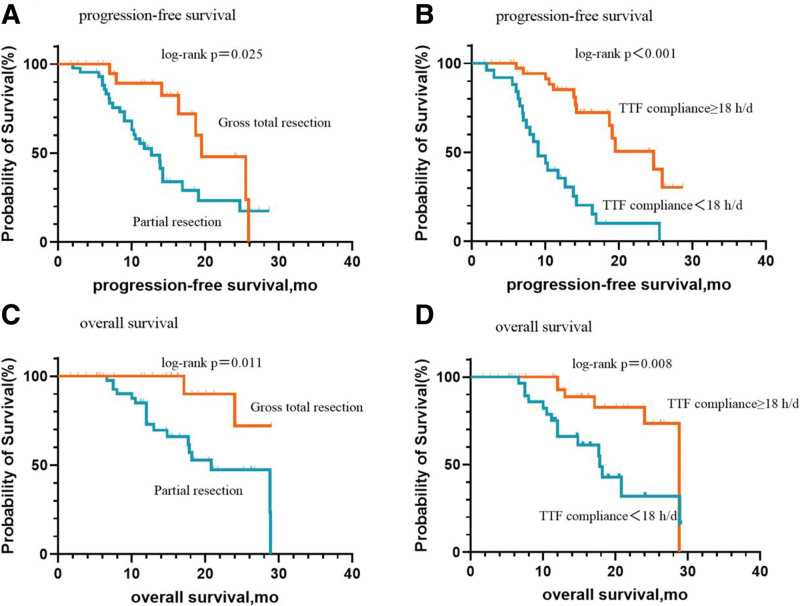
Kaplan–Meier survival analysis in comparing gross total resection versus partial resection, progression-free survival (PFS) is shown in (A) and overall survival (OS) is shown in (C); TTF compliance ≥18 h/d versus <18 h/d, progression-free survival (PFS) is shown in (B) and overall survival (OS) is shown in (D). TTFields, tumour treating fields.

Prognostic factors were determined utilizing the Cox proportional hazards model and are summarized in Tables 2 and 3. Univariate analysis showed that PFS was better after total tumor resection (*P* = .03) and with a compliance rate ≥ 18 h/d (*P* = .00). Multivariate analysis also showed that total tumor resection (*P* = .034; HR = 2.45; 95%CI: 1.07–5.62) (Fig. 3A) was independent prognostic factors for PFS, and with a compliance rate ≥ 18 h/d (*P* **<** .001; HR = 5.39; 95% CI: 2.54–11.44) (Fig. 3B) were independent prognostic factors for PFS. Gender, MGMT methylation status, isocitrate dehydrogenase (IDH) status, 1p19q combined codeletion status, TERT promoter status, and concomitant oral anlotinib treatment had no effect on PFS (Table 2).

**Table 2:** Univariate and multivariate analyses of progression-free survival (PFS) in GBM patients.

Variable	PFS	Numbers of patients	Univariate analysis		Multivariate analysis	
			*P*-value	HR (95% CI)	*P*-value	HR (95% CI)
Sex	Men	37				
	Women	31	.058	1.95 (0.98–3.90)		
Extent of resection	Gross total resection	43				
	Partial resection	25	**.030**	**2.42 (1.09–5.36**)	**.034**	**2.45 (1.07–5.62**)
Anlotinib plus TTFields	Yes	50				
	No	18	.494	0.76 (0.34**–**1.68)		
MGMT promotor region methylationstatus	UnMethylated	20				
	Methylated	23	.350	1.48 (0.65**–**3.38)		
	Unknown	25	.823	0.91 (0.41**–**2.04)		
IDH status	Wild-type	37				
	Mutated	11	.442	1.42 (0.58**–**3.49)		
	Unknown	20	.447	0.63 (0.19**–**2.01)		
TERT promoter mutation status	UnMethylated	11				
	Methylated	20	.519	1.37 (0.53**–**3.59)		
	Unknown	37	.202	1.61 (0.77**–**3.35)		
Tumor tissue chromosomes 1p and 19q	Codeletion	4				
	Retained	21	.604	1.21 (0.59**–**2.47)		
	Unknown	43	.712	1.26 (0.37**–**4.32)		
TTFields compliance	≥18 h/d	25				
	<18h/d	43	**<.001**	**5.19 (2.52–10.70**)	**.000**	**5.39 (2.54–11.44**)

Bold values indicates statistically significant differences (*P* < .05).

GBM = glioblastoma, IDH1 = isocitrate dehydrogenase, MGMT = O6-methylguanine-DNA-methyltransferase gene, TERT = telomerase reverse transcriptase, TTFields = tumor-treating-fields.

**Table 3:** Univariate and multivariate analyses of overall survival (OS) in GBM patients.

Variable	OS	Numbers of patients	Univariate analysis		Multivariate analysis	
			*P*-value	HR (95% CI)	*P*-value	HR (95% CI)
Sex	Men	37				
	Women	31	**.013**	**4.06 (1.35–12.20**)	.051	2.67 (1.00**–**7.24)
Extent of resection	Gross total resection	43				
	Partial resection	25	**.024**	**5.41 (1.25–23.35**)	.063	4.07 (0.92**–**17.92)
Anlotinib plus TTF	Yes	50				
	No	18	.372	0.61 (0.20**–**1.82)		
MGMT promotor region methylationstatus	Unmethylated	20				
	Methylated	23	.147	2.21 (0.76**–**6.45)		
	Unknown	25	.624	0.74 (0.22**–**2.45)		
IDH status	Wild-type	37				
	Mutated	11	.902	0.93 (0.30**–**2.86)		
	Unknown	20	.966	–		
TERT promoter mutation status	Unmethylated	11				
	Methylated	20	.876	0.90 (0.24**–**3.44)		
	Unknown	37	.836	1.11 (0.40**–**3.09)		
Tumor tissue chromosomes 1p and 19q	Codeletion	4				
	Retained	21	.922	1.05 (0.43**–**2.57)		
	Unknown	43	.982	–		
TTF compliance	≥18 h/d	25				
	<18 h/d	43	**.013**	**3.45 (1.30–9.16**)	.066	2.88 (0.93**–**8.89)

Bold values indicates statistically significant differences (*P* < .05).

GBM = glioblastoma, IDH1 = isocitrate dehydrogenase, MGMT = O6-methylguanine-DNA-methyltransferase gene, TERT = telomerase reverse transcriptase, TTFields = tumor-treating-fields.

Univariate analysis showed that women (*P* = .013), total tumor resection (*P* = .024) (Fig. 3C), and with a compliance rate ≥ 18 h/d (*P* = .013) (Fig. 3D) had better OS. Multivariate analysis showed that no independent prognostic factors favorable for OS were observed (Table 3). MGMT methylation status, IDH status, 1p19q codeletion status, TERT promoter status, and concurrent oral anlotinib treatment had no effect on OS (Table 3).

### 3.4. Treatment-related adverse events

**Figure 4.**
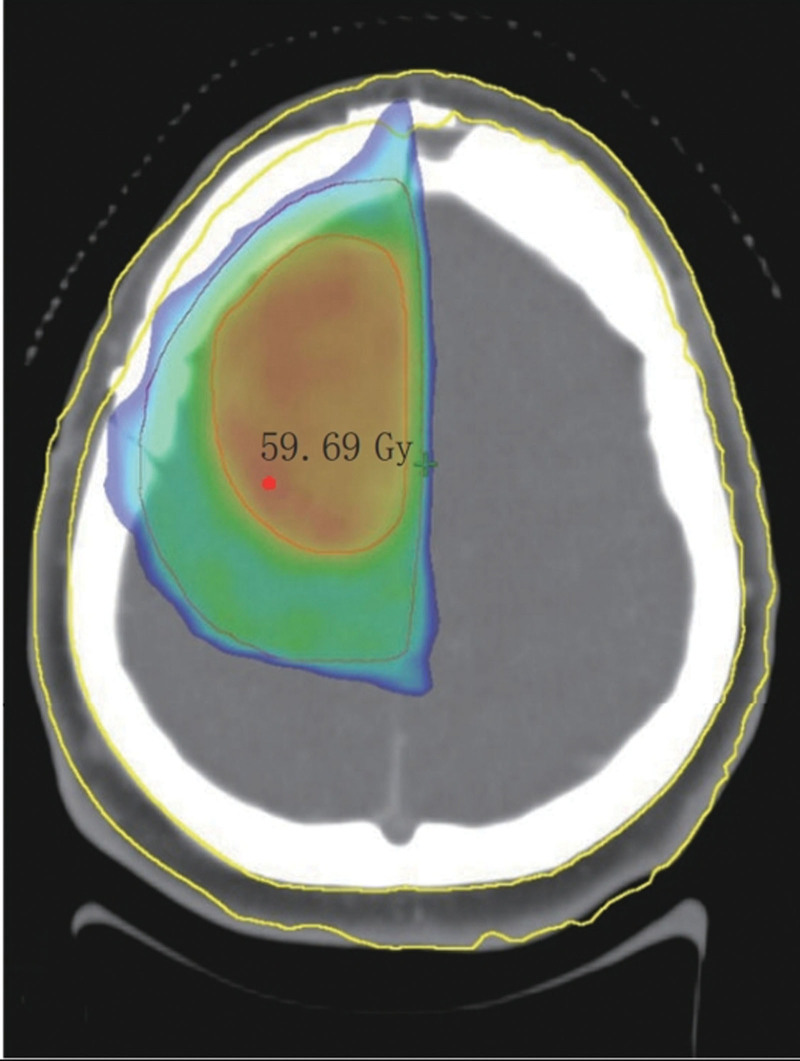
Example of dose distribution in a patient with high-grade glioma (Patient #6).

A scalp-protective radiotherapy plan was implemented for all 68 patients, with a $D_{20\;cm^{3}}$ of 38.83 ± 6.64 Gy. Figure 4 shows the dose distribution in Patient #6. By dose-limiting the scalp, the percentage of the area covered by the 40 Gy dose in the scalp structure was reduced.

Among the 68 patients, 1 (1.5%) had grade 3 leukopenia, 2 (2.9%) had grades 3 to 4 thrombocytopenia, 1 (1.5%) had grade 3 hyperkalaemia, and 1 (1.5%) had neutropenia. The remaining patients with grades 1 to 2 hematological adverse reactions, including leukopenia in 8 patients (11.6%), thrombocytopenia in 4 patients (5.8%), hypokalemia in 6 patients (8.8%), hyperkalaemia in 2 patients (2.9%), hyponatraemia in 7 patients (10.3%), hypernatraemia in 1 patient (1.5%), and hypercalcaemia in 4 patients (5.8%), developed hypercalcaemia, and 2 patients (2.9%) developed hypocalcemia (Table 4).

**Table 4:** Adverse events in the blood system.

Adverse reaction	Number of cases (%)	Adverse reaction	Number of cases (%)
Level 1–2		Hypercalcaemia	4 (5.8)
Leukopenia	8 (11.6)	Hypocalcemia	2 (2.9)
Neutropenia	4 (5.8)	High cholesterol	10 (14.7)
Reduced hemoglobin	4 (5.8)	Elevated alanine aminotransferase	15 (22.1)
Thrombocytopenia	4 (5.8)	Elevated gamma-glutamate transpeptidase	12 (17.6)
Hyperkalaemia	2 (2.9)	Level 3–4	
Hypokalemia	6 (8.8)	Leukopenia	1 (1.5)
Hypernatraemia	1 (1.5)	Thrombocytopenia	2 (2.9)
Hyponatraemia	7 (10.3)	Hypokalemia	1 (1.5)
Hypertriglyceride	5 (7.4)	Neutropenia	1 (1.5)

There were 35 TTFields-related dAEs, with an incidence of 51.4%. These included grade 1 dAEs in 42.6% (29/68 patients) and grade 2 dAEs in 8.8% (6/68 patients), with no grades 3 to 4 dAEs (Table 5). Contact dermatitis was observed in 13 patients (19.1%), blisters in 7 patients (10.3%), lesions or ulcers in 9 patients (13.2%). Four patients with scalp abscesses discontinued TTFields usage for 3 days. After receiving treatment with aggressive topical steroids and antibiotic ointment, the adverse scalp reactions were assessed as grade 1 dAE, and then the wearing of TTFields continued to be resumed, which ensured the effectiveness and consistency of the use of TTFields. Two other patients, due to the large extent of seepage from his scalp abscess, the TTField ceramic piece was unable. The other patient was unable to avoid the area of scalp abscess with the TTField ceramic piece because of the large area of scalp abscess oozing, so he stopped wearing the TTField on the 41st day of simultaneous radiotherapy (42 days in total) and was significantly relieved after 7 days with local treatment of steroid hormone and antibiotic ointment, and the adverse scalp reaction was assessed as grade 1 dAE. No balance disorders, falls or fractures related to the use of therapeutic devices occurred.

**Table 5:** Dermatological adverse events in the skin reactions.

dAE level	Number of cases (%)
Level 1	
Contact dermatitis	19.1 (13/68)
Skin lesions or ulcers	13.2 (9/68)
Blisters	10.3 (7/68)
Level 2	
Abscessus	7.4 (5/68)
Severe infection	1.5 (1/68)

dAE = TTField-related dermatological adverse event.

## 4. Discussion

The annual incidence of GBM is steadily increasing, with a nearly 100% recurrence rate and minimal improvement in survival outcomes despite various treatment approaches. Total doses above 60 Gy, as well as fractionation changes, are considered futile.^[9]^ The highly vascularized nature of GBM makes anti-angiogenic therapy very attractive. Unfortunately, a total of 11 clinical trials have not yet demonstrated its ability to improve OS.^[10]^ In response to these challenges, TTFields was created as promising therapeutic modalities. TTFields is a unique therapeutic tool that has no half-life and therefore can be used continuously and is not associated with systemic adverse effects. TTFields primarily disrupt mitotic spindle microtubule formation, and secondary mechanisms include delaying DNA damage repair, upregulating autophagy, inhibiting migration, inducing antitumour immunity, and disrupting the integrity of the blood–brain barrier.^[4]^ The EF-02 trial in 2002 demonstrated the feasibility and safety of TTFields as a novel therapeutic approach.^[11]^ EF-07, a phase I trial of 10 patients with recurrent GBM, achieved PFS and OS rates that were more than twice the median of those reported in historical control patients^[12]^ and confirmed that TTFields was both effective in vivo and in vitro. EF-11, the first phase III clinical trial on TTFields for recurrent GBM, achieved a median OS of 6.6 months in the TTFields group (vs temozolomide [TMZ] alone: 6.0 months), and its lower systemic toxicity as well as greater cognitive and affective functioning propelled the approval of the U.S. Food Drug Administration for the treatment of incurable or recurrent GBMs.^[13]^ EF-14 combined TTFields with temozolomide for newly diagnosed GBM patients had a median OS of 20.9 months (vs TMZ alone: 16.0 months), a 5-year survival rate of 13% (vs TMZ alone: 5%), and no increase in adverse events but no decrease in (health-related quality of life); these results are undoubtedly important.^[5]^ Moreover, studies have indicated that TTFields and radiotherapy have synergistic effects on common targets and similar mechanisms of action; both can prevent cell migration and invasion and induce mitotic abnormalities and DNA damage.^[6]^ A study by Guberina et al^[14]^ demonstrated for the first time that the concurrent administration of TTFields with radiotherapy is an effective therapeutic strategy. Preclinical studies by Kirson et al^[7]^ and the results of EF-14^[5]^ also confirmed that TTFields can improve the efficacy of chemotherapy without increasing toxicity. Currently, there is a gap regarding the efficacy and safety of TTFields combined with simultaneous chemoradiotherapy for the treatment of newly diagnosed GBM in Chinese patients. Further research in this area is warranted to optimize treatment strategies and improve patient outcomes.

In this study, a total of 68 patients with newly diagnosed GBM were treated with simultaneous radiotherapy and TTFields, 35 of whom experienced progression by the last follow-up, including 20 who died. The median PFS for all patients was 16.4 months, the median OS for all patients was 28.8 months. Survival data were subjected to further follow-up. Notably, in the EF-14 trial,^[5]^ the median PFS in the TTFields with temozolomide combination therapy group was 6.7 months, the median OS was 28.8 months. A prospective study by Bokstein et al^[13]^ included a total of 10 patients with newly diagnosed GBM treated with simultaneous radiochemotherapy, and TTFields showed that the median PFS of all patients was 8.9 months. TTFields was associated with a median PFS of 16.4 months, a result that appears favorable versus historical benchmarks but requires confirmation in propensity-score-matched or randomized cohorts. Prospective randomized trials or well-powered propensity-matched analyses are necessary to confirm any survival advantage attributable to TTFields.

In this study, gross total resection, and a compliance rate ≥80% were independent prognostic factors for PFS. However, the MGMT methylation status, IDH mutation status, 1p19q codeletion status, and TERT promoter status had no effect on PFS. This finding may be attributed to the limited availability of molecular sequencing data, resulting in a notable proportion of missing data for these molecular tests. Multivariate analysis showed that no independent prognostic factors favorable for OS were observed. The follow-up time was short to contribute to it. With the futher follow-up time were prolonged, the issue can be solved.

The EF-14 results showed that compliance with TTFs significantly improved the prognosis of patients with GBM. The 5-year survival rate of GBM patients with a compliance rate ≥90% reached 29.3%.^[5]^ The Patient Registry Dataset recommends that patients should have an adherence ≥75%.^[15]^ In our study, compliance with the daily use of TTFields ≥ 80% was significantly associated with mortality according to univariate analyses. It was a significant predictor of survival time and was consistent with the results of this trial.

Our study is limited by its retrospective, single-center design, and relatively small sample size (n = 68). First, due to the referral nature of our institution, comprehensive molecular profiling was unavailable for some patients; these missing data are classified as “Unknown” rather than definitive negative results. Second, reflecting the transition in diagnostic criteria, we retained 4 patients with 1p/19q codeletion and a subset with unknown IDH status based on their histological diagnosis and standardized treatment. Consequently, strictly applying the 2021 WHO classification suggests our cohort likely includes cases of IDH-mutant astrocytoma (WHO grade 4) and oligodendroglioma. While this introduces heterogeneity and potential prognostic bias, it faithfully represents the real-world patient population managed with this trimodal therapy.

The special point in this study is the use of concurrent oral anlotinib, the ALTER0303 experiment by Jiang et al^[16]^ has found that, anlotinib which is not only active in the brain, but also can play a role in tumor control of brain metastases, improve survival time, and delay intracranial progression. However, concurrent oral anlotinib was not observed to be associated with higher PFS and OS in this study, and its use in the first-line treatment of patients with GBM is still debatable.

Scalp irritation is the most common adverse reaction during treatment with TTFields, so the 68 GBM patients in this trial were treated with a scalp-protective radiotherapy plan. Despite these precautions, scalp adverse reactions, albeit reduced, were still encountered, consistent with findings in the literature. The SPARE study by Miller et al^[17]^ reported that 83.3% of all GBM patients treated with TTFields experienced grades 1 to 2 scalp adverse reactions. In this study, only 53% of patients experienced grades 1 to 2 scalp adverse reactions. Strategies to mitigate the risk of adverse scalp reactions include regular shaving, removing scalp oil, changing the sensor array regularly, and minimizing pressure on the scalp. Timely detection of adverse scalp reactions is crucial, and management strategies involve the use of antiperspirants, steroids, hydrogel dressings, topical antibiotics, and if necessary, oral antibiotics or treatment interruption. The implementation of a scalp-sparing radiotherapy program has further contributed to reducing the incidence of adverse scalp reactions.

Although shaving the scalp is a psychological barrier, chemotherapeutic drugs cause hair loss. Although patients report a lack of social acceptance, patients and families are still able to adapt quickly to the use of the TTFields device. Although many experts have questioned the feasibility and safety of TTFs, numerous clinical trials of TTFs have demonstrated promising prospects. Furthermore, emerging treatments such as lyssavirus, CAR-T-cell therapy, and immunotherapy for GBM have also yielded promising results,^[17]^ with ongoing exploration in several clinical trials. Ultimately, the combination of maximum safe surgery followed by radiotherapy with concurrent temozolomide chemotherapy as well as TTFields represents a novel first-line treatment option for patients with GBM, offering hope for improved outcomes and quality of life.

## Author contributions

**Conceptualization:** Zixuan Wang, Jingshu Xu, Xia He.

**Data curation:** Zixuan Wang, Zhenyuan Jiang, Jingshu Xu.

**Formal analysis:** Zixuan Wang, Zhenyuan Jiang, Yizhi Ge, Xiao Wang.

**Funding acquisition:** Xia He.

**Investigation:** Zixuan Wang, Wenjie Guo.

**Methodology:** Zixuan Wang, Dan Zong, Wenjie Guo.

**Project administration:** Dan Zong, Huanfeng Zhu.

**Resources:** Xia He.

**Software:** Zixuan Wang, Huanfeng Zhu, Guannan Zou, Xia He.

**Supervision:** Zixuan Wang, Dan Zong, Huanfeng Zhu.

**Validation:** Zixuan Wang, Dan Zong, Huanfeng Zhu.

**Visualization:** Zixuan Wang, Dan Zong, Huanfeng Zhu.

**Writing – original draft:** Zixuan Wang.

**Writing – review & editing:** Zixuan Wang.
